# Outcomes of patients treated with venetoclax plus azacitidine versus azacitidine alone stratified by advanced age and acute myeloid leukemia composite model

**DOI:** 10.1038/s41375-025-02730-3

**Published:** 2025-09-05

**Authors:** Adriano Venditti, Jing-Zhou Hou, Pierre Fenaux, Brian A. Jonas, Radovan Vrhovac, Pau Montesinos, Jacqueline S. Garcia, David Rizzieri, Michael J. Thirman, Meng Zhang, Jalaja Potluri, Catherine Miller, Mazaher Dhalla, Vinod Pullarkat

**Affiliations:** 1 Hematology, Department of Biomedicine and Prevention, University Tor Vergata, Rome, Italy; 2 Hematology and BMT, Lemieux Center for Blood Cancers, UPMC Hillman Cancer Center, Pittsburgh, PA USA; 3 Hôpital St. Louis/Assistance Publique‑Hôpitaux de Paris and Université de Paris, Paris, France; 4 Department of Internal Medicine, Division of Malignant Hematology, Cellular Therapy and Transplantation, University of California Davis School of Medicine, Sacramento, CA USA; 5 Department of Haematology, University Hospital Centre Zagreb, and University of Zagreb School of Medicine, Zagreb, Croatia; 6 Hospital Universitari i Politecnic la Fe, Valencia, Spain; 7 Department of Medical Oncology, Dana‑Farber Cancer Institute, Harvard Medical School, Boston, MA USA; 8 Novant Health Cancer Institute, Winston Salem, NC USA; 9 Department of Medicine, Section of Hematology/Oncology, University of Chicago, Chicago, IL USA; 10 AbbVie Inc., North Chicago, IL USA; 11 Department of Hematology and Hematopoietic Cell Transplantation and Gehr Family Center for Leukemia Research, City of Hope Comprehensive Cancer Center, Duarte, CA USA

**Keywords:** Acute myeloid leukaemia, Targeted therapies

## Abstract

Venetoclax plus azacitidine is recognized as standard of care for patients with acute myeloid leukemia (AML) ineligible for intensive chemotherapy (IC). However, some patients may still not be treated with venetoclax combinations due to frailty concerns. We evaluated efficacy and safety of venetoclax plus azacitidine vs. placebo plus azacitidine in patients with newly diagnosed AML ineligible for IC from the phase 3 VIALE-A study (NCT02993523) and the phase 1b M14-358 study (NCT02203773), stratified by two methods to potentially assess frailty. The first method was age-based (75–79, 80–84, ≥85 years; *n *= 303 pooled from both studies) and the second was fitness-based using the AML composite model (AML-CM), a comorbidity-based model to estimate mortality risk (Group A, B, C; *n *= 380, from VIALE-A). Efficacy, including composite complete remission and overall survival, were improved with venetoclax plus azacitidine vs. placebo plus azacitidine across age and AML-CM groups. Safety was generally similar between age and AML-CM groups and no new safety signals were identified. Taken together, these data suggest that patients benefit from venetoclax plus azacitidine regardless of age or degree of frailty and the combination may be considered for patients with AML who may be deemed frail. Clinical trial information NCT02993523; NCT02203773.

## Introduction

Acute myeloid leukemia (AML) is the most common malignant myeloid disorder in adults, with the median age at presentation being 70 years [1]. The 5-year relative survival of patients with AML is ~32%, and survival rates are dependent on age at diagnosis. Patients <50 years of age have an estimated 5-year survival of 63.2%, but this decreases substantially with increasing age (50–64 years, 38.4%; 65–74 years, 18.0%; ≥75 years, 4.2%) [2].

About half of adults with AML do not receive intensive chemotherapy (IC) aimed at inducing remission due to older age or comorbidities. Older patients deemed ineligible for IC often receive less-intensive regimens or supportive/palliative care [3–8]. Although patients offered non-intensive and palliative measures tend to be older, have greater comorbidities, and are considered frail or less fit, the criteria for frailty are not clearly defined [5, 9, 10]. Therefore, more attention must be given to understand frailty, as novel treatment combinations may lead to increased side effects in this vulnerable patient population [11–14].

While advanced age has historically been considered a poor prognostic factor and has been used, along with performance status, comorbidities, and cognitive assessments, to determine patient fitness for IC, there is no standard method for determining frailty [9, 15–17]. Due to the ambiguity of this approach, it is unclear what percentage of patients with AML are frail; published rates vary between 17% and 68% [18–20].

In this analysis, we used two methods to analyze patients who may be deemed frail. First, we stratified patients based on advancing age. Although age alone is not the primary predictor for treatment outcomes, it has been used as a surrogate for other factors, such as comorbidities and performance status, which increase with age [13, 21]. Second, we used the AML composite model (AML-CM), which incorporates age, comorbidities, and cytogenic and/or molecular risks to provide an objective scale to stratify patient fitness [22]. The AML-CM, which has been validated to estimate mortality of patients with AML, was developed to identify older patients with AML who, based on age alone, may have traditionally been offered palliative care but who could benefit from active treatment with intensive chemotherapy.

Venetoclax plus azacitidine is the current standard of care (SOC) for patients with newly diagnosed AML who are not eligible for IC, based on findings from the Phase 3 VIALE-A study. In the full VIALE-A study cohort, venetoclax plus azacitidine treatment was associated with significantly higher composite complete remission (CRc; defined as complete remission [CR] + CR with incomplete hematologic recovery [CRi]) rates (66.4% vs. 28.3%) and longer median overall survival (OS; 14.7 months vs. 9.6 months) compared with placebo plus azacitidine [23]. Long-term follow-up (median, 43.2 months) has demonstrated durable remissions in patients treated with venetoclax plus azacitidine [24]. Despite being recognized as the SOC for all patients ineligible for IC, questions remain as to whether some patients may still be considered too frail to receive venetoclax-based therapy. The goal of this post-hoc analysis is to examine safety, efficacy, and quality of life (QoL) of patients stratified by two methods to identify patients who were more or less likely to be considered frail and determine whether they derived clinical benefit from venetoclax plus azacitidine vs. azacitidine alone.

## Methods

### Patients and study designs

**Fig. 1:**
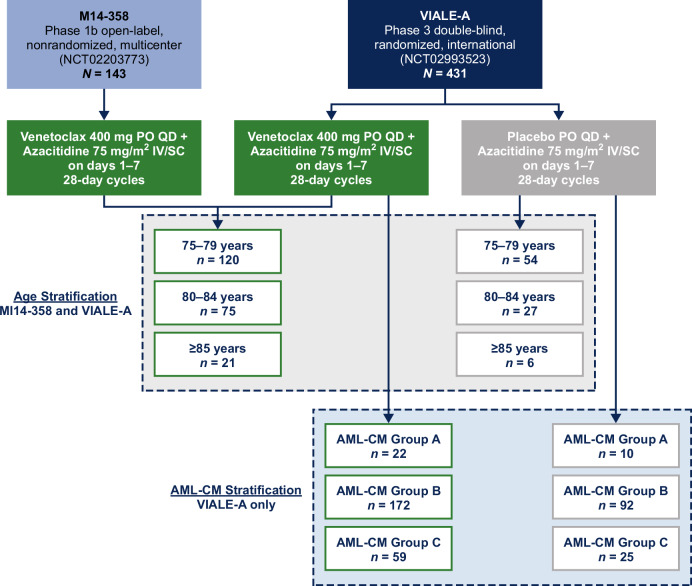
Study design and patient stratification. AML-CM acute myeloid leukemia composite model, IV intravenous, PO oral, QD once per day, SC subcutaneous.

To examine the role of fitness in patient outcomes after venetoclax + azacitidine treatment versus azacitidine alone, two exploratory analyses were conducted. The first stratified patients by age, as age is a predominant factor in determining fitness. The second analysis stratified patients using the AML-CM scale, which describes patient fitness based on age, comorbidities, and cytogenetic/molecular disease factors. For the analysis of patients stratified by advanced age, data were pooled from the phase 3 VIALE-A study (NCT02993523) and the nonrandomized, single-arm phase 1b M14-358 study (NCT02203773); study designs and eligibility criteria have been reported previously (Fig. 1) [23, 25]. Patients included from both studies received 400 mg venetoclax and 75 mg/m^2^ azacitidine and were all deemed ineligible for intensive chemotherapy by the same criteria. Patients were separated into 3 groups: 75–79 years of age, 80–84 years of age, and ≥85 years of age. To analyze patients stratified by fitness defined by AML-CM, only data from patients in the VIALE-A study were used, as the phase 3 trial collected more comprehensive comorbidity data (Fig. 1). AML-CM scores for patients in VIALE-A were calculated as previously described (Supplementary Fig. S1) [22]. Higher scores were associated with worse prognosis and decreased benefit from IC. Group A (AML-CM score 1–4) defined patients who could potentially benefit from IC; Group B (AML-CM score 5–9) those who have decreased benefit from IC; Group C (AML-CM score ≥10) patients who could potentially benefit from a clinical trial or palliative care.

Patients enrolled in both studies had a confirmed diagnosis of AML by World Health Organization (WHO) criteria (VIALE-A, 2016 criteria; M14-358, 2008 criteria), received no prior therapy for AML, and were ineligible for standard IC due to age ≥75 years or comorbidities. Patients enrolled in the VIALE-A study were randomized 2:1 to venetoclax plus azacitidine or placebo plus azacitidine. Patients from the phase 1b study received open-label venetoclax plus azacitidine or decitabine. Patients on both studies received venetoclax at 400 mg orally on days 1–28 after ramp-up and azacitidine at 75 mg/m^2^ intravenously or subcutaneously on days 1–7 every 28-day cycle, or placebo plus azacitidine. In both protocols, patients were treated with 28 days of venetoclax until marrow clearance after which dose adjustments were implemented and growth factors could be used.

### Ethics approval and consent to participate

This post hoc analysis is based on data from two studies that previously received all necessary review board and ethics committee approvals [23, 25]. Both studies were conducted in accordance with the International Conference on Harmonization, Good Clinical Practice guidelines, and the Declaration of Helsinki, as previously described. All patients provided written informed consent. No new ethical approvals were necessary for the current analysis.

### Assessments

Baseline assessments for inclusion in VIALE-A or M14-358 were performed at screening [23, 25], followed by response assessments at the end of cycle 1, and every 3 cycles thereafter; patients were evaluated per modified International Working Group (IWG) response criteria for AML and as previously described [23, 26]. All adverse events (AEs) were graded according to the National Cancer Institute Common Terminology Criteria for Adverse Events version 4.0.

In the analysis by advanced age, measurable residual disease (MRD) was assessed centrally by flow cytometry in patients who achieved CRc, with MRD response defined as 1 or fewer residual leukemic blasts per 1000 leukocytes or 10^−3^ per the ELN guidelines [27]. Samples were collected at baseline from bone marrow aspirates during the clinical assessment, the end of cycle 1, and after every 3 cycles thereafter. Patients who had one negative sample for MRD value below the cutoff at any time in the study were defined as having an MRD response. QoL was assessed with the use of the European Organisation for Research and Treatment of Cancer Quality of Life questionnaire (EORTC QLQ) and the Patient-Reported Measurement Information System (PROMIS) 7A Fatigue questionnaire. For the EORTC, global health status outcomes are reported on a scale of 0–100 with a higher score representing higher/better level of functioning [28, 29]. For PROMIS 7A Fatigue, a mean score of 50 is based on the US population, with higher scores indicating more fatigue [30].

### Statistical analysis

The clinical data cutoff dates were December 1, 2021 for the VIALE-A study and July 19, 2019 for the M14-358 study. Demographics were summarized by descriptive statistics. Remission rates were summarized in counts and proportions, and confidence intervals (CIs) were estimated using the exact binomial method. OS and duration of response were evaluated by the Kaplan–Meier methodology. The hazard ratio (HR) and 95% CI between treatment groups were estimated using the Cox proportional hazards model. Where possible, identical variables were examined across the age-based and fitness-based analyses, but some relevant outcomes that were only reported by the pooled data for one of the analyses were included.

The patient populations in VIALE-A and M14-358 were not powered for statistical comparisons of QoL across age stratification. In this post-hoc analysis, EORTC QLQ and PROMIS 7A Fatigue outcomes were numerically compared to detect patterns associated with frailty.

## Results

### Outcomes to venetoclax plus azacitidine in elderly patients based on age

#### Patient disposition and baseline characteristics

This pooled analysis stratified 216 patients by advanced age from VIALE-A and M14-358 in the venetoclax plus azacitidine cohorts (75–79 years, *n *= 120; 80–84 years, *n *= 75; ≥85 years, *n *= 21) and 87 patients treated with placebo plus azacitidine from the VIALE-A (75–79 years, *n *= 54; 80–84 years, *n *= 27; ≥85 years, *n *= 6) (Fig. 1). Baseline characteristics were largely similar across age groups, including ECOG performance status and cytogenic risk; baseline Grade 3/4 neutropenia was more prevalent in patients ≥85 years of age (Table 1).

**Table 1:** Patient baseline characteristics by age category.

Parameter	Aged 75–79 years		Aged 80–84 years		Aged ≥85 years	
	Ven + Aza (*n* = 120)	Pbo + Aza (*n* = 54)	Ven + Aza (*n* = 75)	Pbo + Aza (*n* = 27)	Ven + Aza (*n* = 21)	Pbo + Aza (*n* = 6)
Median age (range), years	77 (75–79)	77 (75–79)	81 (80–84)	81 (80–84)	86 (85–91)	86 (85–90)
Male sex, *n* (%)	67 (56)	35 (65)	45 (60)	14 (52)	13 (62)	4 (67)
Geographic region, *n* (%)						
United States	30 (25)	8 (15)	24 (32)	3 (11)	6 (29)	2 (33)
Outside of United States	90 (75)	46 (85)	51 (68)	24 (89)	15 (71)	4 (67)
ECOG PS, *n* (%)						
0–1	85 (71)	39 (72)	61 (81)	23 (85)	15 (71)	3 (50)
2–3	35 (29)	15 (28)	14 (19)	4 (15)	6 (29)	3 (50)
Neutropenia at baseline, *n* (%)						
Grade 1–2	15 (13)	11 (20)	8 (11)	5 (19)	2 (10)	1 (17)
Grade 3–4	84 (70)	33 (61)	55 (73)	18 (67)	19 (90)	5 (83)
Cytogenetics, *n* (%)						
Intermediate	77 (67)	31 (57)	47 (66)	19 (70)	14 (70)	3 (50)
Poor	38 (33)	23 (43)	24 (34)	8 (30)	6 (30)	3 (50)
Missing	5	0	4	0	1	0
*TP53* mutated, *n*/*N* (%)	18/73 (25)	7/31 (23)	12/52 (23)	3/21 (14)	3/18 (17)	0/4
Bone marrow blast count, *n* (%)						
<30%^a^	38 (32)	16 (30)	20 (27)	8 (30)	5 (24)	0
30% to <50%	26 (22)	13 (24)	23 (31)	4 (15)	6 (29)	4 (67)
≥50%	56 (47)	25 (46)	32 (43)	15 (56)	10 (48)	2 (33)
Type of AML, *n* (%)						
Primary/de novo	82 (68)	38 (70)	55 (73)	19 (70)	18 (86)	5 (83)
Secondary	38 (32)	16 (30)	20 (27)	8 (30)	3 (14)	1 (17)
AML with myelodysplasia-related changes, *n* (%)	41 (34)	21 (39)	23 (31)	9 (33)	8 (38)	2 (33)

*AML* acute myeloid leukemia, *Aza* azacitidine, *CTCAE* Common Terminology Criteria for Adverse Events, *ECOG PS* Eastern Cooperative Oncology Group performance status, *Pbo* placebo, *Ven* venetoclax.

^a^Patients from VIALE-A had bone marrow blast counts between 20% and 29% [23].

#### Efficacy outcomes

**Fig. 2:**
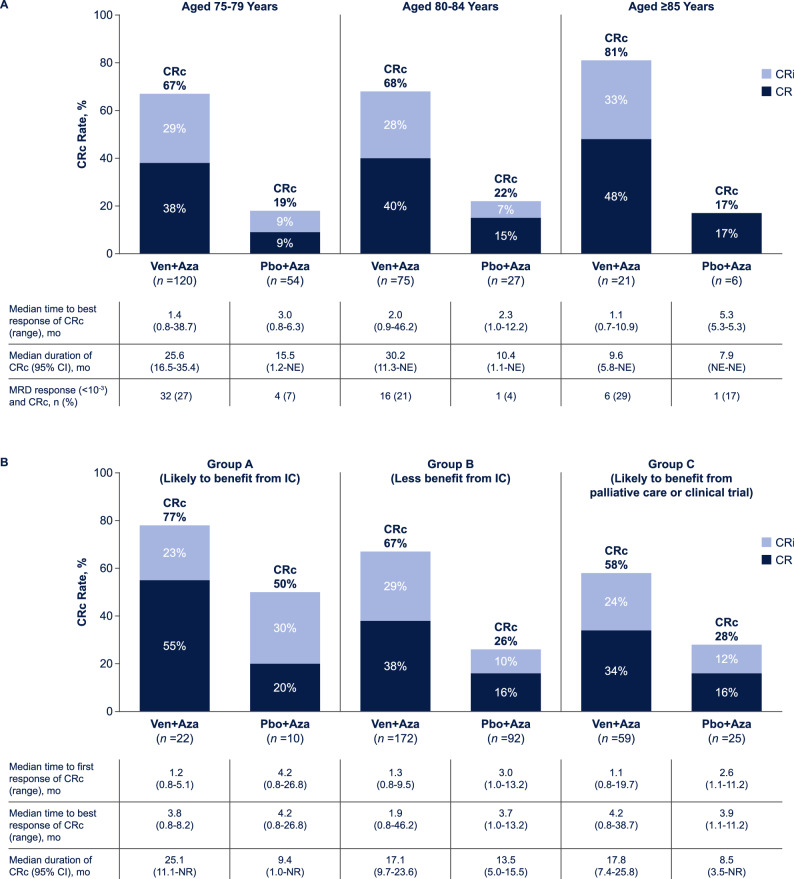
Efficacy outcomes. CRc rates and MRD response rates by age category (**A**) and AML-CM group (**B**). AML-CM acute myeloid leukemia composite model, Aza azacitidine, CR complete remission, CRc complete remission + complete remission with incomplete hematologic recovery, CRi complete remission with incomplete hematologic recovery, MRD measurable residual disease, NE not estimable, Pbo placebo, Ven venetoclax.

CRc rates were higher with venetoclax plus azacitidine vs. placebo plus azacitidine across all age groups (75–79 years, 67% vs. 19%; 80–84 years, 68% vs. 22%; ≥85 years, 81% vs. 17%; Fig. 2A). The median time to best response of CRc was faster (75–79 years, 1.4 months [range, 0.8–38.7] vs. 3.0 months [0.8–6.3]; 80–84 years, 2.0 [0.9–46.2] vs. 2.3 [1.0–12.2]; ≥85 years, 1.1 [0.7–10.9] vs. 5.3 [5.3–5.3]) and median duration of response was longer (75–79 years, 25.6 months [95% CI, 16.5–35.4] vs. 15.5 months [1.2–not estimable (NE)]; 80–84 years, 30.2 [11.3–NE] vs. 10.4 [1.1–NE]; ≥85 years, 9.6 [5.8–NE] vs. 7.9 [NE–NE]) in the venetoclax plus azacitidine group compared with the placebo plus azacitidine group, respectively, across all age groups; however, the number of responding patients were small in some groups, particularly the placebo plus azacitidine older age groups (80–84 years, *n *= 6; ≥85 years, *n *= 1). MRD response rates in CRc responders were higher with venetoclax plus azacitidine compared with placebo plus azacitidine across all age cohorts (75–79 years, 27% vs. 7%; 80–84 years, 21% vs. 4%; ≥85 years, 29% vs. 17%; Fig. 2A).

**Fig. 3:**
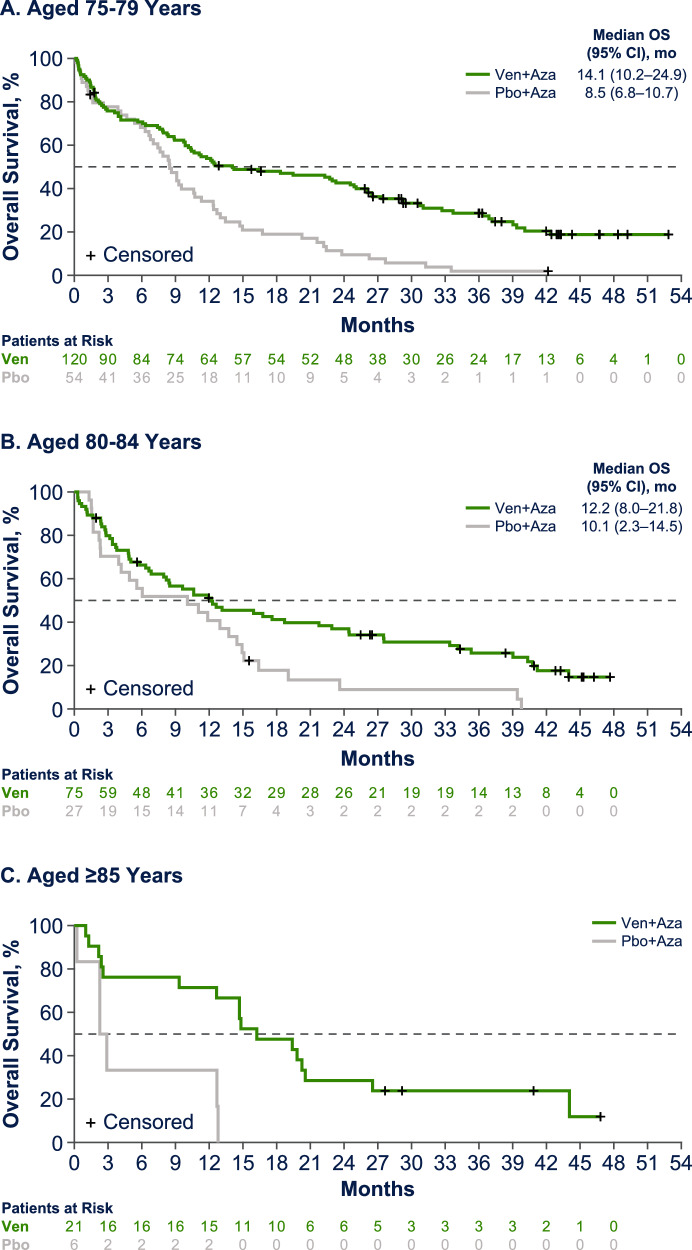
Overall survival by age category. Overall survival for patients aged 75-79 years (**A**), 80-84 years (**B**), and ≥85 years (**C**). Aza azacitidine, NE not estimable, OS overall survival, Pbo placebo, Ven venetoclax.

For patients ≥75 years of age, median OS was 14.1 months (95% CI, 10.7–19.8) in the venetoclax plus azacitidine group and 8.5 months (95% CI, 6.0–10.7) in the placebo plus azacitidine group (Fig. 3), and improvement in median OS with venetoclax plus azacitidine was evident across age groups. In the venetoclax plus azacitidine vs. placebo plus azacitidine arms, respectively, median OS (95% CI) was 14.1 (10.2–24.9) vs. 8.5 (6.8–10.7) months for patients aged 75–79 years, 12.2 (8.0–21.8) vs. 10.1 (2.3–14.5) months for those aged 80–84 years, and 16.2 (9.3–20.5) vs. 2.6 (0.2–NE) patients aged ≥85 years. The proportion of deaths in patients treated with venetoclax plus azacitidine vs. placebo plus azacitidine was 74% (*n *= 89/120) vs. 96% (*n *= 52/54) for patients 75–79 years, 77% (*n *= 58/75) vs. 96% (*n *= 26/27) for patients 80–84 years, and 81% (*n *= 17/21) vs. 100% (*n *= 6/6) for patients ≥85 years (Supplementary Table S1). Rates of transfusion independence were higher in patients treated with venetoclax plus azacitidine vs. placebo plus azacitidine across all age cohorts (Supplementary Table S2).

#### Safety outcomes

The safety analysis set included 213 patients from M14-358 and VIALE-A receiving venetoclax plus azacitidine and 85 patients from VIALE-A receiving placebo plus azacitidine. The median number of venetoclax plus azacitidine treatment cycles was 7.0 (range, 1.0–46.0) for patients 75–79 years, 7.0 (1.0–45.0) for patients 80–84 years, and 8.0 (1.0–41.0) for patients ≥85 years. The median venetoclax dosing duration per cycle was 21.0 days (range, 1.0–39.0) for patients aged 75–79, 21.0 days (range, 1.0–36.0) for those aged 80–84, and 21.0 days (range, 10.0–34.0) for those aged ≥85. The most common grade ≥3 treatment-emergent AEs (TEAEs) by age group are noted in Table 2. In general, hematologic AEs occurred more frequently in the venetoclax plus azacitidine group compared with the placebo plus azacitidine group across all age cohorts.

**Table 2:** Most common grade ≥3 TEAEs (>20%) by age category.

Preferred term, *n* (%)	Aged 75–79 years		Aged 80–84 years		Aged ≥85 years	
	Ven + Aza (*n* = 119)	Pbo + Aza (*n* = 54)	Ven + Aza (*n* = 73)	Pbo + Aza (*n* = 26)	Ven + Aza (*n* = 21)	Pbo + Aza (*n* = 5)
Grade ≥ 3 TEAE^a^	117 (98)	52 (96)	72 (99)	25 (96)	21 (100)	4 (80)
Febrile neutropenia	51 (43)	9 (17)	28 (38)	4 (15)	11 (52)	0
Thrombocytopenia	43 (36)	17 (31)	35 (48)	12 (46)	9 (43)	2 (40)
Anemia	36 (30)	10 (19)	19 (26)	4 (15)	10 (48)	1 (20)
Neutropenia	41 (34)	14 (26)	27 (37)	7 (27)	6 (29)	1 (20)
Pneumonia	29 (24)	14 (26)	26 (36)	7 (27)	7 (33)	0
Hypokalemia	17 (14)	6 (11)	3 (4)	3 (12)	2 (10)	2 (40)

*Aza* azacitidine, *Pbo* placebo, *TEAE* treatment-emergent adverse event, *Ven* venetoclax.

^a^Includes grade ≥3 TEAEs with any-grade occurrence in >25% of patients.

The proportion of patients who discontinued treatment due to TEAEs was similar between the venetoclax plus azacitidine group and the placebo plus azacitidine group across all age cohorts (Supplementary Table S3). Deaths due to TEAEs were similar between the venetoclax plus azacitidine group and the placebo plus azacitidine group across all age cohorts. Pneumonia was the most commonly reported fatal AE in the venetoclax plus azacitidine arm (9 [4%] vs. 2 [2%] in the placebo plus azacitidine group; Supplementary Table S4).

No differences in baseline mean or change from baseline were observed between the venetoclax plus azacitidine group and the placebo plus azacitidine group across age cohorts with regard to the PROMIS 7A Fatigue score (Supplementary Table S5) or EORTC QLQ-C30 global health status score (Supplementary Table S6).

### Outcomes to venetoclax plus azacitidine in patients categorized by AML-CM score

#### Patient disposition and baseline characteristics

Patients from the phase 3 VIALE-A study were stratified based on AML-CM score as an alternative method to understand outcomes in potentially frail patients (full criteria, Supplementary Fig. S1). A total of 253 patients were in the venetoclax plus azacitidine group (Group A, *n* = 22; Group B, *n* = 172; Group C, *n* = 59) and 127 patients in the placebo plus azacitidine group (Group A, *n* = 10; Group B, *n* = 92; Group C, *n* = 25). Consistent with enrollment of patients ineligible for IC in VIALE-A, 91% of patients receiving venetoclax plus azacitidine and 92% of patients receiving placebo plus azacitidine were in Groups B or C, associated with poorer prognostic disease characteristics, adverse ELN risk, and other comorbidities. A total of 51 patients could not be categorized due to missing data.

Baseline characteristics were largely similar across AML-CM groups, including age and ECOG performance status. There were more patients with ELN intermediate and adverse risk and poor cytogenetics in Groups B and C (Table 3). All patients with pre-existing cardiac dysfunction, including ejection factor (EF) < 50% and stable angina at baseline had an AML-CM score of ≥5 and were in Groups B or C.

**Table 3:** Patient baseline characteristics by fitness per AML-CM score.

Parameter	Group A		Group B		Group C	
	Ven + Aza (*n* = 22)	Pbo + Aza (*n* = 10)	Ven + Aza (*n* = 172)	Pbo + Aza (*n* = 92)	Ven + Aza (*n* = 59)	Pbo + Aza (*n* = 25)
Median age (range), years	75 (66–84)	73 (65–79)	77 (49–91)	76.5 (60–90)	74 (65–90)	75 (62–87)
Male sex, *n* (%)	11 (50)	7 (70)	100 (58)	58 (63)	41 (69)	15 (60)
ECOG PS, *n* (%)						
0–1	13 (59)	5 (50)	92 (53)	54 (59)	36 (61)	14 (56)
2–3	9 (41)	5 (50)	80 (47)	38 (41)	23 (39)	11 (44)
Reasons for ineligibility for IC, *n* (%)						
≥75 years of age	13 (59)	5 (50)	109 (63)	56 (61)	29 (49)	11 (44)
18–74 years of age with	9 (41)	5 (50)	63 (37)	36 (39)	30 (51)	14 (56)
ECOG PS 2–3	8 (36)	5 (50)	54 (31)	28 (30)	17 (29)	8 (32)
CHF requiring treatment	0	0	1 (1)	1 (1)	0	2 (8)
Ejection fraction ≤50%	0	0	3 (2)	2 (2)	2 (3)	0
Chronic stable angina	0	0	3 (2)	1 (1)	2 (3)	0
DLCO ≤ 65%	0	0	6 (3)	4 (4)	3 (5)	8 (32)
FEV1 ≤ 65%	0	0	1 (1)	3 (3)	9 (15)	4 (16)
Creatinine clearance ≥30 to <45 mL/min	0	0	4 (2)	4 (4)	6 (10)	1 (4)
Hepatic impairment with total bilirubin >1.5 to ≤3 × ULN	0	0	0	0	3 (5)	0
Other	2 (9)	1 (10)	7 (4)	1 (1)	3 (5)	4 (16)
Cardiac disorders at baseline, *n* (%)						
Atrial fibrillation	0	0	23 (13)	7 (8)	19 (32)	6 (24)
Cardiac failure	0	0	3 (2)	2 (2)	2 (3)	1 (4)
Chronic	0	0	2 (1)	0	3 (5)	0
Congestive	0	0	7 (4)	1 (1)	5 (8)	1 (4)
Coronary artery disease	0	0	11 (6)	5 (5)	15 (9)	2 (8)
Myocardial infarction	0	0	7 (4)	6 (7)	8 (14)	1 (4)
Myocardial ischemia	1 (5)	0	5 (3)	4 (4)	1 (2)	1 (4)
ELN^a^ risk group, *n* (%)						
Favorable	12 (63)	4 (57)	19 (13)	13 (16)	3 (6)	2 (9)
Intermediate	6 (32)	2 (29)	41 (28)	16 (20)	5 (10)	1 (4)
Adverse	1 (5)	1 (14)	86 (59)	52 (64)	43 (84)	20 (87)
Cytogenetics, *n* (%)						
Intermediate	22 (100)	8 (80)	110 (64)	60 (65)	27 (46)	11 (44)
Poor	0	2 (20)	62 (36)	32 (35)	32 (54)	14 (56)
Bone marrow blast count, *n* (%)						
<30%^a^	8 (36)	2 (20)	50 (29)	28 (30)	17 (29)	4 (16)
30% to <50%	5 (23)	3 (30)	31 (18)	17 (18)	14 (24)	9 (36)
≥50%	9 (41)	5 (50)	91 (53)	47 (51)	28 (47)	12 (48)
Type of AML, *n* (%)						
De novo	18 (82)	8 (80)	134 (78)	72 (78)	33 (56)	16 (64)
Secondary	4 (18)	2 (20)	38 (22)	20 (22)	26 (44)	9 (36)
Mutation, *n*/*N* (%)						
*FLT3 ITD* or *TKD*	2/19 (11)	2/7 (29)	19/132 (14)	14/71 (20)	7/44 (16)	6/23 (26)
*IDH1/2*	6/20 (30)	0/8	35/151 (23)	19/83 (23)	16/54 (30)	7/25 (28)
*TP53*	0/11	0/7	30/119 (25)	11/59 (19)	8/34 (24)	1/18 (6)
*NPM1*	6/11 (55)	3/7 (43)	19/119 (16)	13/59 (22)	2/34 (6)	1/18 (6)

Group A (AML-CM score 1–4) were patients who could potentially benefit from IC; Group B (AML-CM score 5–9) were patients who had decreased benefit from IC; Group C (AML-CM score ≥10) were patients who could potentially benefit from a clinical trial or palliative care.

*AML-CM* acute myeloid leukemia composite model, *Aza* azacitidine, *CHF* congestive heart failure, *DLCO* diffusing capacity for carbon monoxide, *ECOG PS* Eastern Cooperative Oncology Group performance status, *ELN* European LeukemiaNet, *FEV1* forced expiratory volume in the first second, *IC* intensive chemotherapy, *Pbo* placebo, *ULN* upper limit of normal, *Ven* venetoclax.

^a^ELN risk groups are based on 2017 criteria.

#### Efficacy outcomes

In the analysis based on AML-CM scores, CRc rates were higher with venetoclax plus azacitidine vs. placebo plus azacitidine across all groups (Group A, 77% vs. 50%; Group B, 67% vs. 26%; Group C, 58% vs. 28%; Fig. 2B). The median time to first response of CRc was faster with venetoclax plus azacitidine vs. placebo plus azacitidine across Groups A (1.2 months [range, 0.8–5.1] vs. 4.2 months [0.8–26.8]), B (1.3 [0.8–9.5] vs. 3.0 [1.0–13.2]), and C (1.1 [0.8–19.7] vs. 2.6 [1.1–11.2]). Median duration of response was longer with venetoclax plus azacitidine compared with placebo plus azacitidine across all groups (Group A, 25.1 months [95% CI, 11.1–NE] vs. 9.4 months [1.0–NE]; Group B, 17.1 [9.7–23.6] vs. 13.5 [5.0–15.5]; Group C, 17.8 [7.4–25.8] vs. 8.5 [3.5–NE]; Fig. 2B).

**Fig. 4:**
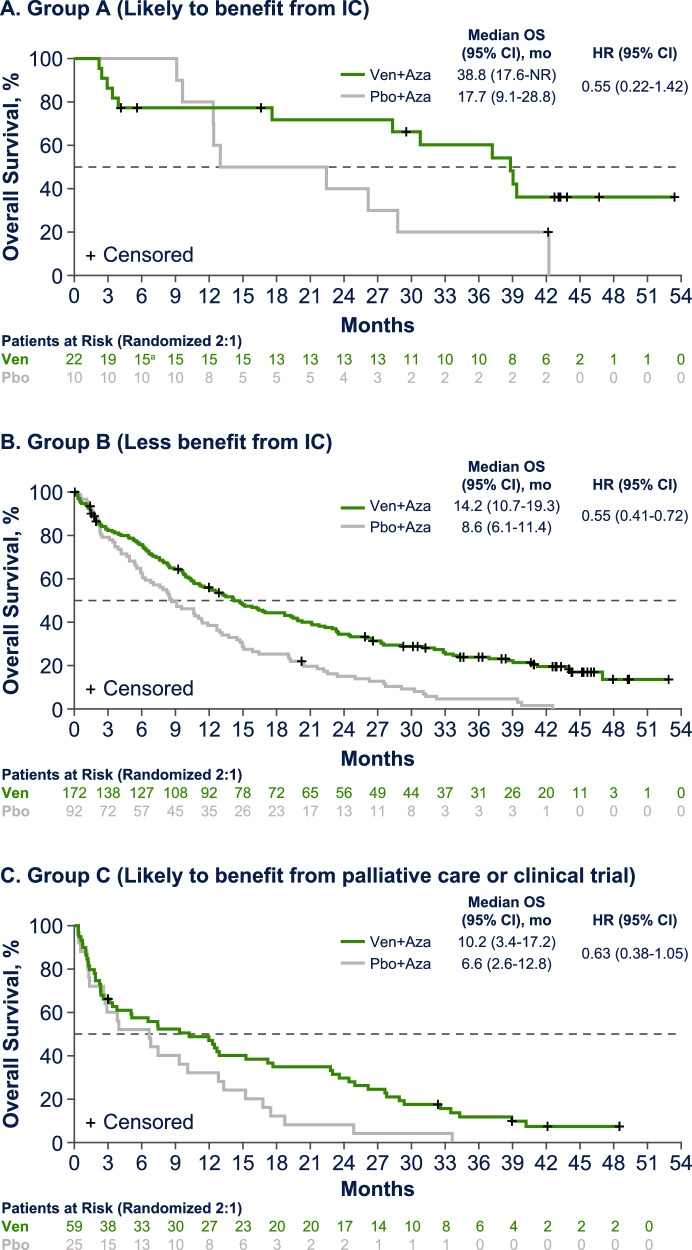
Overall survival by fitness based on AML-CM score. Overall survival for patients likely to benefit from IC (**A**), less likely to benefit from IC (**B**), and likely to benefit from palliative care or a clinical trial (**C**). AML-CM acute myeloid leukemia composite model, IC intensive chemotherapy, Aza azacitidine, NR not reached, OS overall survival, Pbo placebo, Ven venetoclax.

Median OS was longer with venetoclax plus azacitidine vs. placebo plus azacitidine across all groups (Group A, 38.8 months [95% CI, 17.6–NE] vs. 17.7 [9.1–28.8]; Group B, 14.2 [10.7–19.3] vs. 8.6 [6.1–11.4]; Group C, 10.2 [3.4–17.2] vs. 6.6 [2.6–12.8]; Fig. 4). The proportion of deaths in patients treated with venetoclax plus azacitidine vs. placebo plus azacitidine was 54% (*n *= 12/22) vs. 90% (*n *= 9/10) for Group A, 77% (*n *= 133/172) vs. 97% (*n *= 89/92) for Group B, and 90% (*n *= 53/59) vs. 100% (*n *= 25/25) for Group C.

#### Safety outcomes

The safety analysis set included 249 patients receiving venetoclax plus azacitidine and 126 patients receiving placebo plus azacitidine. The median number of venetoclax plus azacitidine treatment cycles was 13.0 (range, 2.0–44.0) for Group A, 7.0 (1.0–45.0) for Group B, and 5.5 (1.0–46.0) for Group C. In the venetoclax plus azacitidine group, the median venetoclax dosing duration per cycle was 23.9 days (range, 16.4–29.0) for Group A, 23.7 days (range, 2.0–30.0) for Group B, and 23.0 days (range, 1.0–29.0) for Group C. In the placebo plus azacitidine group, the median number of treatment cycles was 10.0 (range 1.0–45.0) for Group A, 4.0 (1.0–34.0) for Group B, and 3.0 (1.0–28.0), for Group C, and the median dosing duration per cycle was 27.6 days (range 22.9–28.7) in Group A, 26.8 (2.0–32.2) in Group B, and 24.4 (2.0–29.5) in Group C. The most common grade ≥3 TEAEs by AML-CM score are noted in Table 4. In general, hematologic and gastrointestinal AEs occurred more frequently in patients treated with venetoclax plus azacitidine compared with placebo plus azacitidine across all AML-CM groups.

**Table 4:** Most common grade ≥3 TEAEs (>20%) by fitness per AML-CM score.

Preferred term, *n* (%)	Group A		Group B		Group C	
	Ven + Aza (*n* = 21)	Pbo + Aza (*n* = 10)	Ven + Aza (*n* = 170)	Pbo + Aza (*n* = 91)	Ven + Aza (*n* = 58)	Pbo + Aza (*n* = 25)
Grade ≥ 3 TEAE^a^	21 (100)	9 (90)	169 (99)	88 (97)	56 (97)	24 (96)
Thrombocytopenia	15 (71)	5 (50)	86 (51)	37 (41)	20 (34)	7 (28)
Anemia	9 (43)	2 (20)	48 (28)	19 (21)	11 (19)	2 (8)
Neutropenia	9 (43)	6 (60)	70 (41)	22 (24)	28 (48)	5 (20)
Febrile neutropenia	8 (38)	0	79 (46)	20 (22)	23 (40)	5 (20)
Leukopenia	7 (33)	2 (20)	27 (16)	6 (7)	10 (17)	2 (8)
Pneumonia	6 (29)	3 (30)	51 (30)	24 (26)	23 (40)	10 (40)
Hypokalemia	3 (14)	0	17 (10)	9 (10)	5 (9)	6 (24)

Group A (AML-CM score 1-4) were patients who could potentially benefit from IC; Group B (AML-CM score 5-9) were patients who had decreased benefit from IC; Group C (AML-CM score ≥10) were patients who could potentially benefit from a clinical trial or palliative care.

*AML-CM* acute myeloid leukemia composite model, *Aza* azacitidine, *IC* intensive chemotherapy, *Pbo* placebo, *TEAE* treatment-emergent adverse event, *Ven* venetoclax.

^a^Includes grade ≥3 TEAEs with any-grade occurrence in >25% of patients.

In the safety set (venetoclax plus azacitidine, *n *= 249 and placebo plus azacitidine, *n *= 126) there were 22 deaths within 30 days of the first dose of study drug (Supplementary Table S7). One patient died from disease progression and 21 died due to AEs. All deaths from AEs occurred in patients from Groups B and C which are noted to have higher rates of baseline comorbidities and higher risk of mortality. Of the deaths due to AEs, 16 (6%) occurred in the venetoclax plus azacitidine arm (AML-CM Group B, *n *= 8; AML-CM Group C, *n *= 8) and 5 (4%) in the placebo plus azacitidine arm (AML-CM Group B, *n *= 2; AML-CM Group C, *n *= 3).

## Discussion

Patients who are ineligible for IC are eligible for treatment with venetoclax plus azacitidine, but some patients may not receive treatment due to frailty concerns. Approaches to characterizing patient frailty are still evolving, with no consensus on a definition. Patient fitness for IC is historically established from the Ferrara criteria, but this method does not consider disease characteristics and cytogenetic risk, which are important while considering patient fitness in the context of the disease biology [13, 22]. Results of the VIALE-A study suggest that even patients ≥75 years of age with adverse risk factors may derive greater benefit from novel therapies compared with azacitidine alone. To help better inform clinical decision-making, we used age stratification and the AML-CM score that considers comorbidities and cytogenetic/molecular risk to examine safety and efficacy of venetoclax plus azacitidine in patients who may otherwise be deemed frail and receive suboptimal therapies or only supportive care.

In these post hoc analyses evaluating the efficacy and safety of venetoclax plus azacitidine in patients stratified by advanced age or AML-CM score, venetoclax plus azacitidine was effective regardless of elderly age or AML-CM score. No new safety findings were observed from these post-hoc analyses. Moreover, there was no difference in patient-reported outcomes between treatment arms and across different patient age groups, indicating that the addition of venetoclax did not impair QoL in older patients aged 80–84 years compared with patients aged 75–79 years. Venetoclax plus azacitidine showed higher response rates and longer median OS compared with azacitidine alone across all age cohorts and AML-CM groups. When evaluating by age, improvements in median OS, CRc, and CR were seen in patients ≥85 years of age despite being in the eldest age group, with a median OS of 16.2 months, CRc rate of 81%, and CR rate of 48%. All age groups (≥75 years of age) receiving venetoclax plus azacitidine also consistently fared better in terms of median time to best response of CRc and median duration of response compared with azacitidine alone; however, patients ≥85 years of age had slightly lower duration of CRc (9.6 months) compared with other age-based cohorts. Additionally, QoL measures including fatigue were similar between treatment arms across all age cohorts and transfusion independence rates were higher with venetoclax plus azacitidine vs. azacitidine alone across all age cohorts, despite increases in some AEs with venetoclax. Taken together, these findings suggest that patients should not be excluded from treatment with venetoclax plus azacitidine due to advanced age or age and comorbidities.

In the analysis by AML-CM score, median OS was longer and CRc rates higher across all groups in patients receiving venetoclax plus azacitidine vs. placebo plus azacitidine, with the group considered least fit or frailest having a substantially improved median OS (10.2 months) and CRc (58%) compared with counterparts receiving placebo plus azacitidine (OS: 6.6 months and CRc: 28% respectively). These findings are consistent with previously published data from the overall population of the VIALE-A study showing that treatment with venetoclax plus azacitidine was superior to azacitidine alone in elderly patients or patients ineligible to receive IC [23, 24]. Overall, these data support that if AML induction therapy may be considered in an potentially frail patient, then venetoclax plus azacitidine is preferred to azacitidine alone.

Alternative venetoclax dose schedules for elderly or frail patients with AML was not specifically studied in the context of the VIALE-A or M14-358, however the median duration of venetoclax dosing duration was 21 days (1–42) per cycle among patients (70% age ≥75 years) who had achieved CR/CRi as best response in VIALE-A [24, 31]. The VIALE-A protocol included a 28 day dosing until remission is achieved followed by dose adjustments and G-CSF as clinically required. An ongoing study (NCT03013998) is prospectively assessing alternative venetoclax schedules in patients age 60 years and older [32]. This data may further inform dosing for future therapies including novel triplet combinations utilizing a venetoclax backbone.

In this post-hoc analysis, no additional safety signals were observed. Across both analyses, rates of grade ≥3 TEAEs were generally similar between age and AML-CM groups and were consistent with the known safety profile of venetoclax plus azacitidine [23–25, 33]. While AEs overall were higher with venetoclax plus azacitidine vs. placebo plus azacitidine, rates of discontinuation were similar. Interestingly, in the age-based analysis, patients in the ≥85 years group had broadly similar rates of grade ≥3 TEAEs and TEAEs leading to discontinuation or death compared with patients in the 75–79 years or 80–84 years groups. Further, when patients were stratified by AML-CM score, patients in Group C, considered the frailest cohort per AML-CM score, had generally similar or lower rates of grade ≥3 hematologic TEAEs compared with other AML-CM groups. Although the median venetoclax dosing duration per cycle did not differ between groups based on age or AML-CM stratification, patients in AML-CM Group C received substantially fewer cycles of venetoclax (5.5 cycles) plus azacitidine than patients in Groups B (7.0 cycles) and A (13.0 cycles).

Based on these data, both age-based and AML-CM stratification show longer median OS and higher remission rates with venetoclax plus azacitidine vs. azacitidine alone. Importantly, patients who may be considered the frailest (Group C) derived substantial benefit from venetoclax plus azacitidine, as evidenced by faster, deeper, and more durable remissions compared with placebo plus azacitidine. Taken together, these efficacy and safety results stratified by age, which is a predominant factor in identifying fitness, and by AML-CM scores, which define frailty, suggest that both elderly and patients considered the frailest may still benefit from approved treatment regimens, such as venetoclax plus azacitidine, with manageable safety.

Although AML-CM is a validated model to predict mortality in AML, this objective method was derived from retrospective data which may have limited data informing the model. In addition, the model examined patients treated prior to the approval of venetoclax and had a higher reliance on historic controls such as intensive chemotherapy or HMA monotherapy.

This post hoc analysis was limited by its descriptive nature and the small number of patients in some subgroups, including patients ≥85 years of age. This pooled analysis of patients from the VIALE-A and M14-358 studies was meant to address the limited number of patients in this age group from the VIALE-A study, but the comparison and interpretation of these results warrant caution. Future analyses in large datasets may be needed to further establish the efficacy and safety of venetoclax plus azacitidine in patients ≥75 years of age. In these post-hoc analyses, age and AML-CM groups were used as proxy measures for patient frailty. Despite the limitations of this approach, both measures are readily available in clinical practice and may be incorporated into treatment decision-making. Additional studies examining other measures of frailty and QoL may be important to establish optimal treatment approaches for elderly patients with AML.

In conclusion, venetoclax plus azacitidine demonstrated faster and more durable remissions compared with placebo plus azacitidine regardless of age or fitness based on AML-CM score, with age-stratified patients reporting similar QoL between the two regimens. Results across the subgroups were similar to outcomes reported for the overall population in VIALE-A. These data suggest that venetoclax plus azacitidine, with appropriate monitoring, may be safely considered as a frontline treatment option for older patients with AML despite advanced age or frailty.

## Supplementary information

Supplement

## Data Availability

AbbVie and Genentech are committed to responsible data sharing regarding the clinical trials we sponsor. This includes access to anonymized, individual, and trial-level data (analysis data sets), as well as other information (eg, protocols, clinical study reports, or analysis plans), as long as the trials are not part of an ongoing or planned regulatory submission. This includes requests for clinical trial data for unlicensed products and indications. These clinical trial data can be requested by any qualified researchers who engage in rigorous, independent, scientific research, and will be provided following review and approval of a research proposal, Statistical Analysis Plan (SAP), and execution of a Data Sharing Agreement (DSA). Data requests can be submitted at any time after global approval and after acceptance of this manuscript for publication. The data will be accessible for 12 months, with possible extensions considered. For more information on the process or to submit a request, visit the following link: https://vivli.org/ourmember/abbvie/ then select “Home.”
