# Supplementary material for: Outcomes of patients treated with venetoclax plus azacitidine versus azacitidine alone stratified by advanced age and acute myeloid leukemia composite model
Source: Leukemia. 2025 Sep 5;39(11):2697–707. doi: 10.1038/s41375-025-02730-3 (PMC12589120; doi:10.1038/s41375-025-02730-3)
Supplement: Supplementary file 1 — Supplement [file 41375_2025_2730_MOESM1_ESM.pdf]

Outcomes of Patients Treated With Venetoclax Plus Azacitidine Versus Azacitidine Alone Stratified by Advanced Age and Acute Myeloid Leukemia Composite Model

AUTHORS: Adriano Venditti,[1] Jing-Zhou Hou,[2] Pierre Fenaux,[3] Brian A. Jonas,[4] Radovan Vrhovac,[5] Pau Montesinos,[6] Jacqueline S. Garcia,[7] David Rizzieri,[8] Michael J. Thirman,[9] Meng Zhang,[10] Jalaja Potluri,[10] Catherine Miller,[10] Mazaher Dhalla,[10] Vinod Pullarkat[11]

SUPPLEMENTAL DATA 2

Table S1. Deaths and 30-day overall survival estimate by age category. 2

Table S2. Postbaseline transfusion independence rates by age category. 3

Table S3. TEAEs (≥2 patients) leading to discontinuation by age category. 4

Table S4. TEAEs leading to death by age category. 5

Table S5. PROMIS 7A Fatigue score by age category. 7

Table S6. EORTC QLQ-C30 global health status by age category. 9

Table S7. Early death within 30 days of first dose of study drug by AML-CM fitness group 11

Table S8. CRc rates and MRD response rates by age category and AML-CM group 12

Supplemental Figure S1. AML-CM scoring methodology. 14

SUPPLEMENTAL DATA

Table S1. Deaths and 30-day overall survival estimate by age category.

	**75-79 Years**		**80-84 Years**		**≥85 Years**	
	**Ven+Aza (*n* = 120)**	**Aza (*n* = 54)**	**Ven+Aza (*n* = 75)**	**Aza (*n* = 27)**	**Ven+Aza (*n* = 21)**	**Aza (*n* = 6)**
**Events, deaths, *n* (%)**	89 (74)	52 (96)	58 (77)	26 (96)	17 (81)	6 (100)
**30-day survival estimate, % (95% CI)**	92 (85-95)	89 (77-95)	93 (85-97)	100 (100-100)	100 (100-100)	83 (27-98)

Aza azacitidine, *Ven* venetoclax.

Table S2. Postbaseline transfusion independence rates by age category.

**Postbaseline transfusion independence,[a] *n* (%)**	**75-79 years**		**80-84 years**		**≥85 years**	
	**Ven+Aza (*n* = 120)**	**Pbo+Aza (*n* = 54)**	**Ven+Aza (*n* = 75)**	**Pbo+Aza (*n* = 27)**	**Ven+Aza (*n* = 21)**	**Pbo+Aza (*n* = 6)**
RBC	70 (58)	10 (19)	47 (63)	9 (33)	17 (81)	1 (17)
Platelet	78 (65)	21 (39)	51 (68)	13 (48)	18 (86)	2 (33)
RBC and platelet	68 (57)	10 (19)	46 (61)	8 (30)	16 (76)	1 (17)

Aza azacitidine, *Pbo* placebo, *RBC* red blood cell, *Ven* venetoclax.

[a]Transfusion independence is defined as ≥8 weeks without RBC and/or platelet transfusion.

Table S3. TEAEs (≥2 patients) leading to discontinuation by age category.

**Preferred term, *n* (%)**	**75-79 years**		**80-84 years**		**≥85 years**	
	**Ven+Aza (*n* = 119)**	**Pbo+Aza (*n* = 54)**	**Ven+Aza (*n* = 73)**	**Pbo+Aza (*n* = 26)**	**Ven+Aza (*n* = 21)**	**Pbo+Aza (*n* = 5)**
**Any AE**	**32 (27)**	**11 (20)**	**23 (32)**	**9 (35)**	**6 (29)**	**1 (20)**
Atrial fibrillation	2 (2)	0	2 (3)	0	0	0
Febrile neutropenia	2 (2)	0	2 (3)	0	1 (5)	0
Malignant neoplasm progression	2 (2)	1 (2)	0	2 (8)	0	0
Multiple organ dysfunction syndrome	2 (2)	0	0	0	0	0
Neutropenia	2 (2)	0	0	0	0	0
Pneumonia	2 (2)	1 (2)	2 (3)	2 (8)	0	0
Sepsis	2 (2)	1 (2)	0	1 (4)	2 (10)	0
Systemic inflammatory response syndrome	2 (2)	0	0	0	0	0
Thrombocytopenia	2 (2)	2 (4)	0	0	0	1 (20)
Acute kidney injury	1 (1)	0	2 (3)	1 (4)	0	0
Acute respiratory failure	0	1 (2)	2 (3)	0	0	0
Death	0	0	2 (3)	0	0	0
Spinal compression fracture	0	0	2 (3)	0	0	0

Aza azacitidine, *Pbo* placebo, *TEAE* treatment-emergent adverse event, *Ven* venetoclax.

Table S4. TEAEs leading to death by age category.

Preferred term, *n* (%)	75-79 years		80-84 years		≥85 years	
	**Ven+Aza (*n* = 119)**	**Pbo+Aza (*n* = 54)**	**Ven+Aza (*n* = 73)**	**Pbo+Aza (*n* = 26)**	**Ven+Aza (*n* = 21)**	**Pbo+Aza (*n* = 5)**
**Any AE**	**29 (24)**	**15 (28)**	**20 (27)**	**6 (23)**	**4 (19)**	**1 (20)**
Pneumonia	6 (5)	2 (4)	3 (4)	0	0	0
Sepsis	4 (3)	3 (6)	0	1 (4)	2 (10)	0
Multiple organ dysfunction syndrome	3 (3)	0	0	0	0	0
Hemorrhage intracranial	2 (2)	0	0	0	0	0
Systemic inflammatory response syndrome	2 (2)	1 (2)	0	0	0	0
Atrial fibrillation	1 (1)	0	1 (1)	0	0	0
Candida sepsis	1 (1)	0	0	0	0	0
Cardiac failure	1 (1)	0	0	0	0	0
Cerebrovascular accident	1 (1)	0	0	0	0	0
Cerebral hematoma	1 (1)	0	0	0	0	0
Cerebral hemorrhage	1 (1)	0	0	1 (4)	0	0
Cerebral infarction	1 (1)	0	0	0	0	0
Death	1 (1)	1 (2)	2 (3)	0	0	0
Fungal sepsis	1 (1)	0	0	0	0	0
General physical health deterioration	1 (1)	1 (2)	0	0	1 (5)	0
Klebsiella bacteremia	1 (1)	0	0	0	0	0
Myocardial infarction	1 (1)	0	1 (1)	0	0	0
Sudden death	1 (1)	0	0	0	0	0

Acute myocardial infarction	0	1 (2)	0	0	0	0
Acute respiratory failure	0	1 (2)	2 (3)	0	0	0
Cardiac arrest	0	1 (2)	1 (1)	1 (4)	0	0
Cardio-respiratory arrest	0	1 (2)	0	0	0	0
Cardiovascular insufficiency	0	1 (2)	0	0	0	0
Catheter site hemorrhage	0	1 (2)	0	0	0	0
Escherichia infection	0	1 (2)	0	0	0	0
Klebsiella infection	0	1 (2)	0	0	1 (5)	0
Metabolic acidosis	0	1 (2)	0	0	0	0
Rhinovirus infection	0	1 (2)	0	0	0	0
Sudden cardiac death	0	1 (2)	0	0	0	0
Respiratory failure	0	0	2 (3)	0	0	0
Septic shock	0	0	2 (3)	0	0	0
Anal abscess	0	0	1 (1)	0	0	0
Brain neoplasm	0	0	1 (1)	0	0	0
Cardiovascular disorder	0	0	1 (1)	0	0	0
Escherichia sepsis	0	0	1 (1)	0	0	0
Gastroenteritis salmonella	0	0	1 (1)	0	0	0
Intestinal hemorrhage	0	0	1 (1)	0	0	0
Pneumonia fungal	0	0	1 (1)	0	0	0
Renal failure	0	0	1 (1)	0	0	0
Hemoptysis	0	0	0	1 (4)	0	0
Hypotension	0	0	0	1 (4)	0	0
Pneumonitis	0	0	0	1 (4)	0	0
Enterococcal infection	0	0	0	0	1 (5)	0
Subdural hematoma	0	0	0	0	0	1 (20)

Aza azacitidine, *Pbo* placebo, *TEAE* treatment-emergent adverse event, *Ven* venetoclax.

Table S5. PROMIS 7A Fatigue score by age category.

	75-79 years				80-84 years			
	n	**Baseline mean**	**Within group change vs. baseline, LS mean (SE) [95% CI]**	**Between-group comparison,[a] LS mean diff. (SE) [95% CI]**	***n***	**Baseline mean**	**Within group change vs. baseline, LS mean (SE) [95% CI]**	**Between-group comparison,[a] LS mean diff. (SE) [95% CI]**
**Baseline**								
Pbo+Aza	52	53.60	—	—	24	56.73	—	—
Ven+Aza	89	51.73	—	—	55	52.96	—	—
**Cycle 3**								
Pbo+Aza	33	53.37	-1.22 (1.49) [-4.15, 1.72]	—	12	58.83	-1.19 (2.53) [-6.20, 3.82]	—
Ven+Aza	58	50.65	2.13 (1.13) [-0.10, 4.36]	3.35 (1.85) [-0.28, 6.98]	40	52.08	-3.90 (1.43) [-6.73, -1.07]	-2.71 (2.87) [-8.39, 2.97]
**Cycle 5**								
Pbo+Aza	20	51.82	-0.55 (1.76) [-4.01, 2.91]	—	7	59.31	-7.83 (3.00) [-13.8, -1.90]	—
Ven+Aza	46	49.77	-2.05 (1.22) [-4.45, 0.34]	-1.50 (2.12) [-5.67, 2.66]	31	51.02	-3.40 (1.54) [-6.45, -0.34]	4.44 (3.34) [-2.17, 11.04]
**Cycle 7**								
Pbo+Aza	12	51.43	-3.37 (2.24) [-7.79, 1.04]	—	8	58.95	1.59 (3.02) [-4.39, 7.57]	—
Ven+Aza	47	49.50	-1.29 (1.23) [-3.72, 1.14]	2.09 (2.55) [-2.93, 7.10]	23	53.28	-2.27 (1.73) [-5.69, 1.15]	-3.86 (3.42) [-10.6, 2.9]
**Cycle 9**								

Pbo+Aza	9	54.81	-0.41 (2.66) [-5.65, 4.82]	—	7	59.31	-0.85 (3.17) [-7.12, 5.41]	—
Ven+Aza	36	50.40	-3.82 (1.36) [-6.49, -1.15]	-3.41 (2.97) [-9.25, 2.44]	20	50.67	-3.77 (1.90) [-7.54, 0.00]	-2.92 (3.62) [-10.1, 4.24]
**Cycle 11**								
Pbo+Aza	7	57.94	-3.05 (3.07) [-9.09, 2.99]	—	6	58.08	0.51 (3.39) [-6.21, 7.23]	—
Ven+Aza	35	50.14	-2.31 (1.40) [-5.07, 0.45]	0.74 (3.35) [-5.86, 7.34]	17	53.18	-2.94 (2.07) [-7.04, 1.16]	-3.45 (3.87) [-11.1, 4.21]
**Cycle 13**								
Pbo+Aza	5	60.68	-2.59 (3.56) [-9.59, 4.41]	—	—	—	—	—
Ven+Aza	34	50.00	-2.00 (1.45) [-4.84, 0.86]	0.60 (3.82) [-6.91, 8.11]	—	—	—	—
**Cycle 19**								
Pbo+Aza	5	56.58	-5.46 (3.72) [-12.8, 1.85]	—	—	—	—	—
Ven+Aza	18	52.53	-0.70 (1.86) [-4.36, 2.96]	4.76 (4.11) [-3.32, 12.84]	—	—	—	—

PROMIS 7A Fatigue scores are based on a population mean score of 50, with higher scores indicating more fatigue (Cella, 2016).

Aza azacitidine, *diff* difference, *LS* least squares, *Pbo* placebo, *PROMIS* Patient-Reported Outcome Measurement Information System, *SE* standard error, *Ven* venetoclax.

[a]Compared with Pbo+Aza.

Table S6. EORTC QLQ-C30 global health status by age category.

	75-79 years				80-84 years			
	n	**Baseline mean**	**Within group change vs. baseline, LS mean (SE) [95% CI]**	**Between-group comparison,[a] LS mean diff. (SE) [95% CI]**	***n***	**Baseline mean**	**Within group change vs. baseline, LS mean (SE) [95% CI]**	**Between-group comparison,[a] LS mean diff. (SE) [95% CI]**
**Baseline**								
Pbo+Aza	51	55.39	—	—	23	50.72	—	—
Ven+Aza	89	54.12	—	—	54	56.17	—	—
**Cycle 3**								
Pbo+Aza	33	58.33	2.00 (3.19) [-4.27, 8.28]	—	11	49.24	8.33 (5.11) [-1.79, 18.4]	—
Ven+Aza	58	58.62	2.00 (2.42) [-2.77, 6.77]	-0.01 (3.91) [-7.71, 7.69]	39	60.26	13.3 (2.83) [7.71, 18.9]	4.98 (5.79) [-6.48, 16.4]
**Cycle 5**								
Pbo+Aza	19	60.53	5.46 (3.97) [-2.35, 13.3]	—	7	52.38	1.72 (6.20) [-10.5, 14.0]	—
Ven+Aza	45	60.74	8.07 (2.66) [2.82, 13.3]	2.60 (4.70) [-6.65, 11.9]	30	61.39	14.7 (3.14) [8.48, 20.9]	13.0 (6.86) [-0.62, 26.6]
**Cycle 7**								
Pbo+Aza	11	64.39	13.8 (4.99) [4.01, 23.7]	—	8	52.08	-3.48 (5.88) [-15.1, 8.16]	—
Ven+Aza	47	61.52	3.00 (2.62) [-2.17, 8.16]	-10.84 (5.59) [-21.8, 0.16]	22	54.92	11.53 (3.57) [4.46, 18.6]	15.0 (6.75) [1.65, 28.4]
**Cycle 9**								

Pbo+Aza	9	66.67	9.29 (5.43) [-1.40, 20.0]	—	7	52.38	16.0 (6.20) [3.75, 28.3]	—
Ven+Aza	36	60.65	10.85 (2.89) [5.16, 16.5]	1.56 (6.10) [-10.5, 13.6]	20	56.67	17.3 (3.78) [9.79, 24.8]	1.27 (7.12) [-12.8, 15.4]
**Cycle 11**								
Pbo+Aza	7	66.67	9.09 (6.05) [-2.8, 21.0]	—	6	52.78	6.01 (6.60) [-7.06, 19.1]	—
Ven+Aza	35	61.19	7.38 (2.92) [1.63, 13.1]	-1.71 (6.66) [-14.8, 11.4]	17	59.80	14.3 (4.00) [6.40, 22.2]	8.31 (7.58) [-6.70, 23.3]
**Cycle 13**								
Pbo+Aza	5	61.67	3.79 (6.95) [-9.90, 17.5]	—	—	—	—	—
Ven+Aza	34	61.03	6.11 (2.95) [0.29, 11.9]	2.32 (7.50) [-12.4, 17.1]	—	—	—	—
**Cycle 19**								
Pbo+Aza	5	71.67	12.77 (6.99) [-0.98, 26.53]	—	—	—	—	—
Ven+Aza	18	54.63	1.93 (3.77) [-5.5, 9.36]	-10.84 (7.88) [-26.4, 4.67]	—	—	—	—

EORTC QLQ-C30 global health outcomes are reported on a scale of 0–100 with a higher score representing higher/better level of functioning.

Aza azacitidine, *diff* difference, *EORTC* European Organisation for Research and Treatment of Cancer, *LS* least squares, *Pbo* placebo, *QLQ-C30* Core Quality of Life questionnaire, *SE* standard error, *Ven* venetoclax.

[a]Compared with Pbo+Aza.

Table S7. Early death within 30 days of first dose of study drug by AML-CM fitness group.

	Group A		Group B		Group C	
**Preferred term, *n* (%)**	**Ven+Aza (*n* = 21)**	**Pbo+Aza (*n* = 10)**	**Ven+Aza (*n* = 170)**	**Pbo+Aza (*n* = 91)**	**Ven+Aza (*n* = 58)**	**Pbo+Aza (*n* = 25)**
**Death occurring ≤30 days after first dose of study drug**	0	0	9 (5)	2 (2)	8 (14)	3 (12)
Due to disease progression	0	0	1 (1)	0	0	0
Not due to disease progression	0	0	8 (5)	2 (2)	8 (14)	3 (12)
Unknown	0	0	0	0	0	0

AML-CM acute myeloid leukemia composite model, *Aza* azacitidine, *Pbo* placebo, *Ven* venetoclax.

Table S8. CRc rates and MRD response rates by age category and AML-CM group.

	**75-79 years**		**80-84 years**		**≥85 years**	
	**Ven+Aza (*n* = 120)**	**Pbo+Aza (*n* = 54)**	**Ven+Aza (*n* = 75)**	**Pbo+Aza (*n* = 27)**	**Ven+Aza (*n* = 21)**	**Pbo+Aza (*n* = 6)**
**CR rate, n (%)**	45 (38)	5 (9)	30 (40)	4 (15)	10 (48)	1 (17)
**CRi rate, n (%)**	35 (29)	5 (9)	21 (28)	2 (7)	7 (33)	0
**CR + CRi rate, n (%)**	80 (67)	10 (19)	51 (68)	6 (22)	17 (81)	1 (17)
**Median time to best response of CRc (range), mo**	1.4 (0.8-38.7)	3 (0.8-6.3)	2.0 (0.9-46.2)	2.3 (1.0-12.2)	1.1 (0.7-10.9)	5.3 (5.3-5.3)
**Median duration of CRc (95% CI), mo**	25.6 (16.5-35.4)	15.5 (1.2-NE)	30.2 (11.3-NE)	10.4 (1.1-NE)	9.6 (5.8-NE)	7.9 (NE-NE)
**MRD response (<10^{-3}) and CRc, n (%)**	32 (27)	4 (7)	16 (21)	1 (4)	6 (29)	1 (17)
	**Group A**		**Group B**		**Group C**	
	**Ven+Aza (*n* = 22)**	**Pbo+Aza (*n* = 10)**	**Ven+Aza (*n* = 172)**	**Pbo+Aza (*n* = 92)**	**Ven+Aza (*n* = 59)**	**Pbo+Aza (*n* = 25)**
**CR rate, n (%)**	12 (55)	2 (20)	66 (38)	15 (16)	20 (34)	4 (16)
**CRi rate, n (%)**	5 (23)	3 (30)	50 (29)	9 (10)	14 (24)	3 (12)
**CR + CRi rate, n (%)**	17 (77)	5 (50)	116 (67)	24 (26)	34 (58)	7 (28)
**Median time to first response of CRc (range), mo**	1.2 (0.8-5.1)	4.2 (0.8-26.8)	1.3 (0.8-9.5)	3.0 (1.0-13.2)	1.1 (0.8-19.7)	2.6 (1.1-11.2)
**Median time to best response of CRc (range), mo**	3.8 (0.8-8.2)	4.2 (0.8-26.8)	1.9 (0.8-46.2)	3.7 (1.0-13.2)	4.2 (0.8-38.7)	3.9 (1.1-11.2)
**Median duration of CRc (95% CI), mo**	25.1 (11.1-NR)	9.4 (1.0-NR)	17.1 (9.7-23.6)	13.5 (5.0-15.5)	17.8 (7.4-25.8)	8.5 (3.5-NR)

AML-CM acute myeloid leukemia composite model, *Aza* azacitidine, *CR* complete remission, *CRc* complete remission + complete remission with incomplete hematologic recovery, *CRi* complete remission with incomplete hematologic recovery, *MRD* measurable residual disease, *NE* not estimable, *NR* not reached, *Pbo* placebo, *Ven* venetoclax.

Supplemental Figure S1. AML-CM scoring methodology.

Group A	Group B	Group C
Score 1-4<br>Patients could potentially benefit from IC	Score 5-9<br>Patients have decreased benefit from IC	Score ≥10<br>Patients could potentially benefit from a clinical trial or palliative care

**Component**		**Score**
**Arrhythmia**	Atrial fibrillation, atrial flutter, supraventricular tachycardia, sick sinus syndrome, heart block, ventricular arrhythmia, other	1
**Cardiovascular function**	Coronary artery disease, congestive heart failure, ejection fraction, shortening fraction (for pediatrics)	1
**Inflammatory bowel disease**	Crohn's disease, ulcerative colitis	1
**Cerebrovascular disease**	Transient ischemic attack, subarachnoid hemorrhage, cerebral thrombosis, cerebral embolism, cerebral hemorrhage	1
**Psychiatric disease**	Depression, anxiety, other	1
**Hepatic function**	Total bilirubin/AST/ALT/ALK (×ULN), hepatitis B, hepatitis C, liver cirrhosis or fibrosis	1 or 3
**Obesity**	Calculated BMI (adjusted for age if ≤18 years old)	1
**Infection**	Documented infection, fever of unknown origin, pulmonary nodules suspicious of fungal pneumonia, PPD positive requiring TB prophylaxis, other	1
**Rheumatologic disease**	Systematic lupus erythematosus; rheumatoid arthritis, polymyositis, mixed connective tissue disease; polymyalgia rheumatica, other	2
**Peptic ulcer**	Gastric ulcer, duodenal ulcer	2
**Renal comorbidity**	Creatinine clearance, serum creatinine, on dialysis, prior renal transplantation	2
**Pulmonary score**	DLCO, FEV1, shortness of breath on slight activity, shortness of breath at rest, oxygen supplementation	2 or 3

**Prior solid tumor**		1
**Heart valve disease**	Valve stenosis, valve insufficiency, prosthetic valve, symptomatic mitral valve prolapse	3
**Hypoalbuminemia**		1
**Elevated LDH**		1 or 2
**Age**	<50 years	0
	50-59 years	1
	≥60 years	2
**ELN 2017 risk group**	Favorable	0
	Intermediate	1
	Adverse	3

ALK alkaline phosphatase, *ALT* alanine aminotransferase, *AML-CM* acute myeloid leukemia composite model, *AST* aspartate aminotransferase, *BMI* body mass index, *DLCO* diffusing capacity for carbon monoxide, *ELN* European LeukemiaNet, *FEV1* forced expiratory volume in the first second, *IC* intensive chemotherapy, *LDH* lactate dehydrogenase, *PPD* purified protein derivative, *TB* tuberculosis, *ULN* upper limit of normal.
